# CRF-R1 Antagonist Treatment Exacerbates Circadian Corticosterone Secretion under Chronic Stress, but Preserves HPA Feedback Sensitivity

**DOI:** 10.3390/pharmaceutics13122114

**Published:** 2021-12-08

**Authors:** Yadira Ibarguen-Vargas, Samuel Leman, Rupert Palme, Catherine Belzung, Alexandre Surget

**Affiliations:** 1 UMR1253, iBrain, Université de Tours, Inserm, 37200 Tours, France; yadira.ibarguen-vargas@univ-orleans.fr (Y.I.-V.); samuel.leman@univ-tours.fr (S.L.); 2 EUK-CVL, Université d’Orléans, 45100 Orléans, France; 3 Department of Biomedical Sciences/Biochemistry, University of Veterinary Medicine, 1210 Vienna, Austria; Rupert.Palme@vetmeduni.ac.at

**Keywords:** CRF, CRF receptor type 1 antagonist, HPA axis, glucocorticoids, chronic stress, antidepressant, anxiety, depression

## Abstract

Despite promising initial reports, corticotropin-releasing factor receptor type-1 (CRF-R1) antagonists have mostly failed to display efficacy in clinical trials for anxiety or depression. Rather than broad-spectrum antidepressant/anxiolytic-like drugs, they may represent an ‘antistress’ solution for single stressful situations or for patients with chronic stress conditions. However, the impact of prolonged CRF-R1 antagonist treatments on the hypothalamic–pituitary–adrenal (HPA) axis under chronic stress conditions remained to be characterized. Hence, our study investigated whether a chronic CRF-R1 antagonist (crinecerfont, formerly known as SSR125543, 20 mg·kg^−1^·day^−1^ ip, 5 weeks) would alter HPA axis basal circadian activity and negative feedback sensitivity in mice exposed to either control or chronic stress conditions (unpredictable chronic mild stress, UCMS, 7 weeks), through measures of fecal corticosterone metabolites, plasma corticosterone, and dexamethasone suppression test. Despite preserving HPA axis parameters in control non-stressed mice, the 5-week crinercerfont treatment improved the negative feedback sensitivity in chronically stressed mice, but paradoxically exacerbated their basal corticosterone secretion nearly all along the circadian cycle. The capacity of chronic CRF-R1 antagonists to improve the HPA negative feedback in UCMS argues in favor of a potential therapeutic benefit against stress-related conditions. However, the treatment-related overactivation of HPA circadian activity in UCMS raise questions about possible physiological outcomes with long-standing treatments under ongoing chronic stress.

## 1. Introduction

The identification of pharmacological targets capable of tuning hypothalamic–pituitary–adrenal (HPA) axis activity has been at the forefront of many research and development strategies against chronic stress, anxiety, and depression over the past decades. A promising anticipated strategy has been to target the ACTH-secretagogue CRF (corticotropin-releasing factor). Indeed, (1) CRF release is the primary step toward HPA axis activation and glucocorticoid releases; (2) the CRF system exhibits alterations in depressed patients including higher CRF expressions in the brain [1,2,3,4]; (3) the CRF system also involves forebrain modules, including amygdala and prefrontal cortex [5,6], which can coordinate behavioral responses to stress-associated stimuli and activate hypothalamic neurons upstream to HPA axis; and (4) intracerebral CRF administration or overexpression in rodents induce physiological and behavioral phenotypes that are reminiscent of anxiety and depressive disorders [7]. Taken together, these results indicate that CRF dysfunctions may drive symptomatology and HPA axis abnormalities in stress-related disorders and that glucocorticoid excess may be the downstream reverberation of an altered CRF system.

Within this framework, research efforts converged to develop CRF receptor antagonists, focusing mainly on the CRF receptor type 1 (CRF-R1) that mediates ACTH releases and promotes anxiety-related behaviors in forebrain areas [8]. Preclinical works provided encouraging results, as CRF-R1 antagonists reduced anxiety-like behaviors and induced antidepressant-like effects in diverse rodent models [9,10,11,12,13,14,15,16]. However, despite high translational expectations and an initial promising result [17], all subsequent clinical trials failed to successfully complete double-blind, placebo-controlled studies for anxiety and depressive disorders, mainly for adverse liver effects or lack of efficacy; see [18] for review.

It is, therefore, critical to reassess the medical scopes of the CRF-R1 antagonists and to define realistic avenues for their potential therapeutic uses in stress-related conditions. The most consistent outcome from the preclinical data and from their mechanism of action is their potent ‘antistress’ properties. Accordingly, rather than broad-spectrum antidepressant- or anxiolytic-like drugs, they may represent an ‘antistress’ solution for single stressful situations or for patients with conditions that result precisely from stress-CRF system dysfunctions. Although preclinical studies already demonstrated that a single administration of CRF-R1 antagonists acutely attenuates stress-induced HPA axis activation and improves stress-coping strategies [18], the impact on the HPA axis of prolonged CRF-R1 antagonist treatments under chronic stress conditions remains to be characterized.

We, therefore, aimed to examine the effects of a chronic treatment with the CRF-R1 antagonist crinecerfont on HPA axis basal, circadian activity, and negative feedback sensitivity in mice exposed to a 7-week unpredictable chronic mild stress (UCMS) through measures of fecal corticosterone metabolites, plasma corticosterone, and dexamethasone suppression tests. Crinercefont (formerly known as SSR125543) is a CRF-R1 antagonist that has been subjected to many studies since its first publications in 2002 [10]. In rodent models, it has been shown to decrease anxiety-like behaviors in conflict tests, passive coping strategy in inescapable stress, depression-like state, aggression in social interaction tests, and stress-induced cognitive deficits in rodent models [10,15,19,20,21,22,23,24,25,26,27,28], although it presumably failed to demonstrate efficacy on major depression in a clinical trial (NCT01034995) [18,29]. Acute injection of crinercerfont can interfere with HPA axis activity as several studies demonstrated that it reduces releases of ACTH and corticosterone induced by acute stress in rodents [23,30,31]. However, no study has reported its effects on the HPA axis following chronic administration yet. The UCMS model consists of the chronic exposure to mild socio-environmental stressors inducing physical, behavioral, and neuroendocrine alterations reminiscent of depressive disorders [27,32]. Previous studies showed that chronic treatments with crinecerfont improve physical and behavioral alterations in UCMS [10,15,22,26,27,28], but none of these studies determined the outcomes of such chronic treatments on the HPA axis under chronic stress conditions.

## 2. Materials and Methods

### 2.1. Animals

Male BALB/cByJ mice (Janvier, Le-Genest-Saint-Isle, France) were used, aged 3–4 months, and kept under standard laboratory conditions at their arrival (22 ± 2 °C, 12 h light/dark cycle with lights on at 23:00, food and water ad libitum, housed 4–5 per cage). The animal experiments were conducted following the European Commission Council directive 2010/63/EU, approved by the local ethical committee and French authorities, and complied with ethical guidelines and 3Rs.

### 2.2. Experimental Design

**Figure 1:**
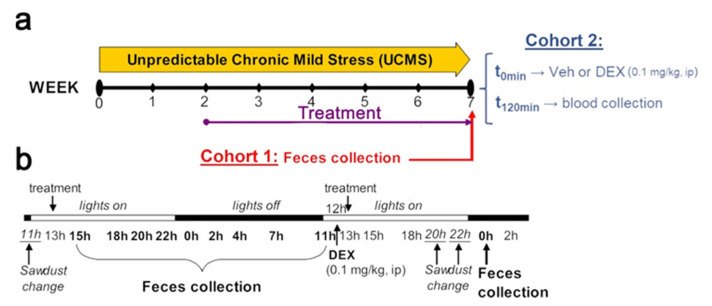
Schematic representation of the experimental design. (**a**) BALB/cByJ mice were exposed to either a 7-week unpredictable chronic mild stress (UCMS) procedure or control conditions. Treatment with either a CRF-R1 antagonist (crinecerfont, 20 mg·kg^−^^1^·day^−^^1^, ip) or vehicle (5% DMSO, 5% Cremophor) starting after two weeks of UCMS and for a total of 5 weeks at the time of either fecal sample collection (cohort 1) or blood collection (cohort 2). (**b**) Detailed time schedule of the fecal sample collection and the dexamethasone (DEX) administration in cohort 1. Nine time points over a day were used to assess baseline HPA axis circadian activity. For the dexamethasone suppression test, 12 h separated the DEX-P injection and the fecal sample collection.

Mice were exposed to either control or unpredictable chronic mild stress (UCMS) conditions for 7 weeks (Figure 1a). From the third week of UCMS onward, mice were administered intraperitoneally (ip) once a day, with either vehicle (5% DMSO, 5% cremophor) or crinecerfont (20 mg·kg^−^^1^·day^−^^1^). This dose was chosen as (1) it represents a crinecerfont concentration that was previously shown to reverse the behavioral effects of UCMS following chronic treatment (3 weeks or more) [15,27,28] and (2) this dose is in a range of concentrations (10–30 mg/kg) from which acute crinecerfont administrations were shown to inhibit ACTH and corticosterone releases to stress [23,31].

At the end of the UCMS, feces were collected from cohort 1 by blind experimenters at nine time points covering 24 h (Figure 1b) for assessing HPA axis circadian activity, as well as a last sample collection the next day (00:00) following a dexamethasone intraperitoneal injection (0.1 mg/kg in 0.9% NaCl) made 12 h earlier for the dexamethasone suppression test. This dexamethasone concentration was chosen based on previous studies showing that this dose significantly reduces subsequent corticosterone releases in BALB/cByJ mice [28,33]. The levels of fecal corticosterone metabolites were used to assess HPA axis circadian activity, while corticosterone suppression (%) was used for dexamethasone suppression test to assess HPA axis negative feedback sensitivity (by comparing levels of fecal corticosterone metabolites at 00:00 without dexamethasone versus at 00:00 following dexamethasone). At the end of the UCMS, mice from cohort 2 were intraperitoneally administered with vehicle (0.9% NaCl) or dexamethasone (0.1 mg/kg) in the dark phase of the light/dark cycle (2 and 4 h following light off), and then were euthanized and blood was immediately collected in order to assess plasma corticosterone at basal levels and following dexamethasone.

Sample size was calculated to detect at least an effect size of η^2^ = 0.175 at α = 0.05 and a statistical power (1-β) = 0.80. In cohort 1, forty-one mice were used at the start of the experiment (*n*_total_ = 41) and randomly assigned to the four experimental groups. However, modest injury at the site of injection was considered as exclusion criteria, resulting in the exclusion of four mice from the experiment as such: control-vehicle (*n* = 11), control-crinecerfont (*n* = 9), UCMS-vehicle (*n* = 9), UCMS-crinecerfont (*n* = 8). In cohort 2, fifty-six mice were used and randomly assigned to the eight experimental groups (*n* = 7 per group): control-vehicle-vehicle, control-vehicle-dexamethasone, control-crinecerfont-vehicle, control-crinecerfont-dexamethasone, UCMS-vehicle-vehicle, UCMS-vehicle-dexamethasone, UCMS-crinecerfont-vehicle, UCMS-crinecerfont-dexamethasone.

### 2.3. Unpredictable Chronic Mild Stress (UCMS)

UCMS mice were single housed in individual cages (24 × 11 × 12 cm) and repeatedly subjected to various socio-environmental stressors according to an unpredictable schedule for a period of 7 weeks (Figure 1), as previously described [32,34]. Control mice were single housed in larger cages (42 × 28 × 18 cm) with plastic tunnels and shelter.

### 2.4. Fecal Sample Collection

For each fecal collection, the litter of the cage was collected and substituted with new sawdust. The feces were then extracted from the litter in another room, which allowed a minimal disturbance of the animals during collection. Each sample thus contained the feces emitted between two time points. The animals were handled several times each day from the onset of the experiment to familiarize them with this procedure.

### 2.5. Fecal Corticosterone Metabolite Enzyme Immunoassay

The fecal samples were dried and homogenized. Aliquots of 0.05 g were extracted with 1 mL of 80% methanol. Extracted fecal samples were then analyzed for immunoreactive corticosterone metabolites using a 5α-pregnane-3β,11β,21-triol-20-one enzyme immunoassay (EIA), as previously described [35,36]. This home-bred EIA has been developed and fully validated for mice; for details, see [35,36]. Concentrations of fecal corticosterone metabolites reflect adrenocortical activity well [37]. Although fecal corticosterone metabolite levels cannot provide a temporal resolution as precise as plasma corticosterone levels, the major advantage of collecting feces is to circumvent important methodological bias induced by invasive procedures to allow multiple 24 h time points per animal and to comply with higher ethical standards [37].

### 2.6. Plasma Corticosterone Radioimmunoassay

Mice were killed by CO_2_ asphyxiation 2 h after dexamethasone injection, and then decapitated. The trunk blood was immediately collected, plasma separated, and stored at −20 °C until corticosterone radioimmunoassay. Plasma was analyzed for total corticosterone levels using a ^125^I-labeled corticosterone double-antibody radioimmunoassay kit (MP Biomedicals, Santa Ana, CA, USA) according to the manufacturer’s protocol and as done before [28]. To avoid inter-assay variability, all the samples were duplicated and run in a single assay (the intra-assay variability was 4.5%). According to the manufacturer, the assay sensitivity was 7.7 ng/mL and the percentage of cross-reactivity with steroids was corticosterone 100%, desoxycorticosterone 0.34%, testosterone 0.1%, cortisol 0.05%, aldosterone 0.03%, progesterone 0.02%, androstenedione 0.01%, 5α-dihydrotestosterone 0.01%, and others < 0.01%.

### 2.7. Statistics

Each individual mouse was considered as the statistical unit. ANOVAs were carried out for (1) 24 h baseline circadian activity based on fecal corticosterone metabolites using “environment” (control, UCMS) and ”treatment” (vehicle, crinecerfont) as categorical factors, as well as ”time point” as repeated measures; (2) dexamethasone suppression test based on fecal corticosterone metabolites using “environment” (control, UCMS) and ”treatment” (vehicle, crinecerfont) as categorical factors; and (3) plasma corticosterone levels using “environment” (control, UCMS), “treatment” (vehicle, crinecerfont), and “dexamethasone” (vehicle, dexamethasone) as categorical factors. Main factor or interaction significant effects (α < 0.05) were followed up with multiple planned comparisons using Holm–Bonferroni correction.

## 3. Results

### 3.1. Fecal Corticosterone Metabolites

#### 3.1.1. HPA Axis Circadian Activity

**Figure 2:**
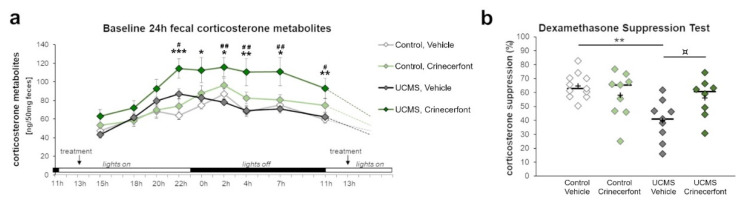
The CRF-R1 antagonist crinecerfont treatment increased circadian fecal corticosterone levels and preserved HPA axis negative feedback under chronic stress. The baseline circadian activity of the HPA axis (panel **a**) and the dexamethasone suppression test (panel **b**) were assessed using the levels of fecal corticosterone metabolites. (**a**) The 5-week crinecerfont treatment (20 mg·kg^−1^·day^−1^, ip) exacerbated the level of corticosterone metabolites at six out of nine time points over a day, uniquely in mice exposed to the unpredictable chronic mild stress (UCMS). *n* = 8–11 per group; * *p* < 0.05, ** *p* < 0.01, and *** *p* < 0.001 UCMS-crinecerfont vs. control-vehicle mice; ^#^ *p* < 0.05 and ^##^ *p* < 0.01 UCMS-crinecerfont vs. UCMS-vehicle mice. Data represent mean ± sem. (**b**) The dexamethasone suppression test allowed assessing the integrity of the HPA axis negative feedback. UCMS significantly decreased the ability of corticosterone suppression in vehicle mice, an effect that tended to be cancelled with crinecerfont treatment. *n* = 8–11 per group; ¤ *p* < 0.10 and ** *p* < 0.01 between line-connected groups. Scatterplot represents individual results, with the ‘+’ symbol showing the mean and the bar representing the median.

We collected fecal samples from the cage of each mouse representing nine time points of fecal boli production spanning 24 h (Figure 1), and enabling us to picture the circadian variations of HPA axis activity in each experimental group (Figure 2a). Our results revealed significant effects of “time point” (F_8,296_ = 26.798; *p* < 0.0001), “environment” (F_1,37_ = 7.231; *p* < 0.05), “treatment” (F_1,37_ = 13.699; *p* < 0.001), as well as interactions of “time point–environment” (F_8,296_ = 2.786; *p* < 0.01), “time point–treatment” (F_8,296_ = 2.353; *p* < 0.05), and “environment–treatment” (F_1,37_ = 4.405; *p* < 0.05). Comparing UCMS-vehicle to control-vehicle mice, we did not identify main significant impacts of UCMS all along the circadian activity of HPA axis. It is, however, worth mentioning that the circadian peak was attained at 22:00 in the UCMS mice before the onset of the dark phase, contrary to control non-stressed mice that attained their peak at 02:00, during the first quarter of the dark phase, suggesting an advanced circadian phase in UCMS-exposed mice. While crinecerfont did not affect HPA axis circadian activities in control non-stressed mice, we found a significant effect of crinecerfont in UCMS mice, reflecting environment–treatment interactions. Crinecerfont resulted in a strong increase in fecal corticosterone metabolite concentrations in UCMS mice all along the circadian cycle, reaching significancy at five and six time points out of nine according to planned pairwise comparisons with UCMS-vehicle mice (22:00, 02:00, 04:00, 07:00, 11:00) and control-vehicle mice (22:00, 00:00, 02:00, 04:00, 07:00, 11:00), respectively (Figure 2a). It is noteworthy that the UCMS-induced shift of the circadian peak (advanced phase) was maintained in crinecerfont-treated mice.

#### 3.1.2. HPA Axis Negative Feedback

We also assessed the effects of UCMS and treatment on HPA axis negative feedback sensitivity through a dexamethasone suppression test (Figure 2b). For this purpose, we injected mice with dexamethasone (0.1 mg/kg, ip) at 12:00 and collected the fecal boli produced between 22:00 and 00:00 (Figure 1b). Our results revealed significant “environment” effects (F_1,33_ = 8.812; *p* < 0.01) and “environment–treatment” interaction (F_1,33_ = 6.536; *p* < 0.05). Compared with control-vehicle mice, UCMS induced a significant reduction of the corticosterone suppression in vehicle-treated mice (Figure 2b). This effect was not found in crinecerfont-treated UCMS mice. Indeed, these mice did not differ from control non-stressed mice, and the treatment tended to improve corticosterone suppression in UCMS mice, suggesting that chronic treatments with CRF-R1 antagonists can reverse HPA axis feedback dysfunctions under chronic stress. 

### 3.2. Plasma Corticosterone

**Figure 3:**
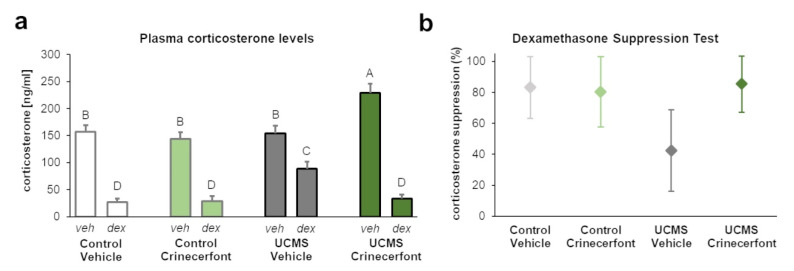
The CRF-R1 antagonist crinecerfont treatment increased basal plasma corticosterone levels and improved HPA axis negative feedback under chronic stress. Plasma corticosterone levels were collected during dark phase and 2 h following vehicle (veh, 0.9% NaCl) or dexamethasone (dex, 0.1 mg/kg, ip) in order to assess basal corticosterone levels and post-dexamethasone corticosterone levels, respectively. (**a**) The 5-week crinecerfont treatment (20 mg·kg^−1^·day^−1^, ip) increased the basal levels of plasma corticosterone uniquely in mice exposed to the unpredictable chronic mild stress (UCMS). UCMS resulted in higher plasma corticosterone levels following dexamethasone administration, an effect reversed by crinecerfont treatment. *n* = 7 per group; the groups not sharing the same letter (A, B, C or D) were found to be significantly different (*p* < 0.05). Data represent mean ± sem. (**b**) Confidence intervals (95%) of the dexamethasone-induced suppression of plasma corticosterone levels computed from data of panel (**a**). The results revealed lower corticosterone suppression levels in UCMS-vehicle mice compared with other groups. *n* = 7 per group.

To confirm the previous results, we examined plasma corticosterone at basal levels and after dexamethasone in another cohort (Figure 3). Our results revealed a significant “environment” effect (F_1,48_ = 19.663; *p* < 0.0001), “dexamethasone” effect (F_1,48_ = 225.099; *p* < 0.0001), “treatment–dexamethasone” interaction (F_1,48_ = 11.568; *p* < 0.01), and “environment–treatment–dexamethasone” interaction (F_1,48_ = 18.263; *p* < 0.0001). As for fecal corticosterone metabolites, we did not identify any significant differences in basal plasma corticosterone levels between control-vehicle and UCMS-vehicle mice, while crinecerfont induced a significant increase in basal plasma corticosterone levels in UCMS mice, but not in control mice (Figure 3a). Dexamethasone injection resulted in a significant decrease in plasma corticosterone levels in the four groups. However, the decrease was significantly attenuated in UCMS-vehicle mice, which display higher post-dexamethasone plasma corticosterone levels compared with the other groups. Consequently, UCMS resulted in a marked decrease of corticosterone suppression in the dexamethasone suppression test in vehicle-treated mice, while crinecerfont treatment restored higher levels of corticosterone suppression in UCMS mice (Figure 3b).

## 4. Discussion

Our study aimed at investigating whether a 5-week-long treatment of a CRF-R1 antagonist would alter HPA axis circadian activity and negative feedback sensitivity in mice exposed to either (1) control or (2) chronic stress conditions. Our findings indicate that a chronic treatment with the CRF-R1 antagonist crinecerfont induces ambivalent influences on HPA axis under chronic stress. Indeed, despite preserving HPA axis circadian activity and negative feedback in control non-stressed mice, the 5-week CRF-R1 antagonist treatment improved the HPA axis negative feedback sensitivity in chronically stressed mice, but paradoxically exacerbated their corticosterone secretion nearly all along the circadian cycle. Considering that altered negative feedback is one of the main signatures of HPA axis dysfunctions in anxiety/depression and that its improvement can precede remission in treatment-responsive patients [38], the capacity of chronic crinecerfont treatments to improve the HPA axis negative feedback in UCMS argues in favor of a potential therapeutic benefit against stress-related conditions. However, the treatment-related overactivation of HPA axis circadian activity in UCMS mice raises questions about possible physiological outcomes with long-standing treatments for patients subjected to ongoing chronic stress conditions.

We found that the UCMS model did not alter the levels of circadian corticosterone secretion, but resulted in a pronounced negative feedback insensitivity and in a modest shift of the circadian phase (earlier corticosterone peak). These results are consistent with previous findings obtained in chronic stress models that similarly revealed an advanced circadian phase [28] and a negative feedback disruption following stress reactivity or dexamethasone suppression tests [28,39,40].

We found a lack of crinecerfont effects on HPA readouts in control non-stressed conditions, which confirms the previous results demonstrating that basal and circadian corticosterone secretions are not altered by subchronic or chronic treatments with other CRF-R1 antagonists including R121919, CP-154,526, and NBI-34041 in both rodents [41,42,43] and humans [17,44,45]. These results suggest that chronic treatments of CRF-R1 antagonists (at doses for which acute administration is effective to inhibit ACTH release) would neither hamper HPA axis activity nor induce adrenal insufficiency in non-stressed conditions. On the other hand, we found that the 5-week CRF-R1 antagonist treatment in UCMS mice (1) improves the HPA axis negative feedback sensitivity, (2) exacerbates their basal circadian corticosterone secretion, and (3) does not normalize the advance phase shift of the HPA axis circadian activity. First of all, the restoration of an operative HPA axis negative feedback by regular antidepressant treatments was previously shown in the UCMS model and is thought to be relevant for clinical effects [28,38], supporting the idea that crinecerfont shares effects that are relevant for antidepressant or antistress effects under chronic stress conditions. This result is interesting as it speaks in favor of a potential therapeutic value of this class of compounds under chronic stress conditions. As acute traumatic stress conditions have also been shown to induce long-lasting HPA axis alterations in stress-susceptible mice [46], it would be interesting to test whether antistress properties of CRF-R1 antagonist treatment might be beneficial for protracted HPA axis alteration induced by acute traumatic stress.

On the other hand, the crinecerfont effects on HPA axis circadian activity are rather intriguing. Indeed, the fact that a CRF-R1 antagonist treatment elicits higher corticosterone circadian secretions in chronic stress conditions may seem counterintuitive when considering its mechanism of action (i.e., blocking the CRF-R1 receptors responsible for activating HPA axis and triggering downstream glucocorticoid release). However, these effects may rely on homeostatic regulations of receptor expression and signaling pathways. Indeed, long-term administration of antagonists induces lower receptor activity and may in return cause receptor/signaling sensitization [47,48,49]. In our study, this mechanism may have resulted in homologous sensitizations within or downstream CRF-R1 signaling as well as in heterologous sensitizations for receptor/signaling associated with other ACTH-secretagogues like arginine-vasopressin (AVP) and catecholamines [50,51,52,53]. As chronic stress can increase AVP and catecholamine releases [54,55,56], their potential receptor/signaling sensitization by CRF-R1 antagonist treatment would potentially result in higher corticosterone secretions in UCMS mice. Such a mechanism would not depreciate HPA axis feedback sensitivity, as negative feedback is mostly triggered by glucocorticoid receptors, or even might strengthen it if heterologous sensitization touches glucocorticoid receptors too.

In summary, our findings demonstrate in two independent cohorts that 5-week treatments with the CRF-R1 antagonist crinecerfont improve the HPA axis negative feedback sensitivity in chronically stressed mice, but paradoxically exacerbate their basal circadian corticosterone secretion. These results point out a marked pharmacodynamic drug–environment interaction on two distinct components of HPA axis activity and suggest considering stress conditions to evaluate the effects of such a medication. Future studies will thus have to identify the biological substrates and regulatory mechanisms causing these effects and to investigate the potential physiological outcomes of long-standing treatments for patients subjected to ongoing chronic stress conditions. It will be particularly valuable as crinecerfont is currently under evaluation for the treatment of classic congenital adrenal hyperplasia, an autosomal recessive disorder that is characterized by impaired corticosteroid synthesis and excess androgens A first report showed beneficial effects on hormone markers following a subchronic 14-day crinecerfont treatment in 18 patients [57]. Based on our results, it will be critical to carefully examine the persistence of such beneficial effects with longer treatments along with life stress assessments in patients. Future studies will also have to evaluate the therapeutic potential of the CRF-R1 antagonist as an ‘antistress’ solution for ad-hoc stressful situations or for patients with conditions that originate precisely from stress-CRF system dysfunctions.

## Data Availability

The datasets generated during and/or analysed during the current study are available from the corresponding author on reasonable request.
